# Genetically programmed retinoic acid deficiency during gastrulation phenocopies most known developmental defects due to acute prenatal alcohol exposure in FASD

**DOI:** 10.3389/fcell.2023.1208279

**Published:** 2023-06-16

**Authors:** B. Petrelli, A. Oztürk, M. Pind, H. Ayele, A. Fainsod, G. G. Hicks

**Affiliations:** ^1^ Department of Biochemistry and Medical Genetics, Regenerative Medicine Program, Faculty of Medicine, University of Manitoba, Winnipeg, MB, Canada; ^2^ Department of Developmental Biology and Cancer Research, Institute for Medical Research Israel–Canada, Faculty of Medicine, Hebrew University of Jerusalem, Jerusalem, Israel

**Keywords:** gastrulation, retinoic acid, vitamin A deficiency, fetal alcohol spectrum disorder (FASD), prenatal alcohol exposure (PAE), craniofacial malformations, malocclusion

## Abstract

Fetal Alcohol Spectrum Disorder (FASD) arises from maternal consumption of alcohol during pregnancy affecting 2%–5% of the Western population. In *Xenopus laevis* studies, we showed that alcohol exposure during early gastrulation reduces retinoic acid (RA) levels at this critical embryonic stage inducing craniofacial malformations associated with Fetal Alcohol Syndrome. A genetic mouse model that induces a transient RA deficiency in the node during gastrulation is described. These mice recapitulate the phenotypes characteristic of prenatal alcohol exposure (PAE) suggesting a molecular etiology for the craniofacial malformations seen in children with FASD. *Gsc* ^*+/Cyp26A1*^ mouse embryos have a r*educ*ed RA domain and expression in the developing frontonasal prominence regi*on* and delayed *HoxA1* and *HoxB1* expression at E8.5. These embryos also show aberrant neurofilament expression during cranial nerve formation at E10.5 and have significant FASD sentinel-like craniofacial phenotypes at E18.5. *Gsc* ^*+/Cyp26A1*^ mice develop severe maxillary malocclusions in adulthood. Phenocopying the PAE-induced developmental malformations with a genetic model inducing RA deficiency during early gastrulation strongly supports the alcohol/vitamin A competition model as a major molecular etiology for the neurodevelopmental defects and craniofacial malformations seen in children with FASD.

## 1 Introduction

Fetal Alcohol Spectrum Disorder (FASD) is the complex neurodevelopmental disorder caused by maternal consumption of alcohol during pregnancy. FASD is very common, affecting approximately 2%–5% of the population in Western societies (Popova et al., 2016; May et al., 2018). FASD with Sentinel Facial Features (Fetal Alcohol Syndrome, FAS), is the most severe form of FASD with an incidence of 7.7 individuals in 1,000 people worldwide (Lange et al., 2017). There is currently no early and simple diagnosis, treatment or cure for FASD. Alcohol dosage, duration of the exposure, whether acute or chronic, and gestational timing are important determinants in the induction and severity of FASD (Godin et al., 2010a; Anthony et al., 2010). Maternal and offspring genetics and epigenetics (Kaminen-Ahola et al., 2010), maternal nutrition (Keen et al., 2010) and metabolism (Burd et al., 2012), and maternal and offspring stress (Weinberg, 1989; Alberry and Singh, 2016) can contribute to the severity of FASD. Presently, FASD diagnosis includes neurobehavioral examination and maternal alcohol intake history (Cook et al., 2016; Hoyme et al., 2016). The clinical categorization of FASD involves its subdivision based on the presence or absence of Sentinel Facial Features that are unique craniofacial malformations present in cases with more severe brain and neurobehavioral anomalies (Chudley et al., 2005; Cook et al., 2016).

In order to study a complex disorder like FASD, animal models that can replicate many phenotypes of the disorder, preferably with minimal variability, high penetrance and reproducibility are of great importance. Ideally, the experimental model should be amenable to testing a developmental hypothesis explaining the FASD etiology. We have previously shown in a *Xenopus laevis* FAS model, that a single “binge” exposure to alcohol during early gastrulation is sufficient to induce developmental defects, including Sentinel Facial Features, characteristic of FASD (Yelin et al., 2005; Yelin et al., 2007). Acetaldehyde from ethanol clearance overwhelms the retinaldehyde dehydrogenases (Aldh1a) present in the embryo that would normally oxidize retinaldehyde to retinoic acid (RA) (Kot-Leibovich and Fainsod, 2009; Shabtai et al., 2018). We have shown that ethanol exposure reduces RA levels during the critical developmental stage of early gastrulation (Shabtai et al., 2018), and we propose that this aberration induces the craniofacial malformations associated with FAS. Ethanol clearance and RA biosynthesis can share the same alcohol and aldehyde dehydrogenases (Shabtai et al., 2018). Alcohol exposure promotes a shift to the removal of impending teratogens, ethanol and acetaldehyde, at the expense of RA biosynthesis. Unfortunately, RA is required during early embryogenesis to regulate numerous developmental processes including the gastrulation process itself, head formation, and neural crest cell patterning (Gur and Bendelac-Kapon, 2022; Gur and Edri, 2022).

Retinoic acid—an active metabolite of Vitamin A—is a diffusible lipophilic molecule that regulates over 3% of genes in the mammalian genome through RA Response Elements (RAREs) and many indirect downstream RA targets (Balmer and Blomhoff, 2002; Paschaki et al., 2013). Analysis of Vitamin A-deficient animal models demonstrated that all-*trans* RA (ATRA) regulates anteroposterior (AP) patterning of the neural plate (Maves and Kimmel, 2005; Sirbu and Duester, 2006). Furthermore, RA is required for migration of neural crest cells from the hindbrain to the frontonasal prominence and pharyngeal arches (Vermot et al., 2003; Rhinn and Dolle, 2012). RA levels are tightly controlled by synthesizing and regulating enzymes like Aldh1a2 (Raldh2), Rdh10 and Dhrs3, and catabolizing cytochrome P450 enzymes like Cyp26A1, Cyp26B1, and Cyp26C1, that modify RA into polar metabolites (4-hydroxy- and 4-oxo-RA), rendering them biologically inactive (Ross and Zolfaghari, 2011; Parihar et al., 2021). Cyp26A1, in particular, metabolizes ATRA, 9-*cis*-RA, and 13-*cis*-RA isomers to prevent inappropriate signaling in certain cell populations or embryonic regions like the developing forebrain (Pennimpede et al., 2010).

The “Alcohol/RA competition model” predicts that RA signaling perturbation at critical developmental stages and regions should induce FASD-like phenotypes (Fainsod et al., 2020). Aldh1a2 (Raldh2) deficient mouse embryos show the extreme effects of Vitamin A (RA) deficiency resulting in embryonic lethality (Niederreither et al., 1999; 2000; 2003). These Aldh1a2 deficient embryos exhibit cell death and abnormal craniofacial neural crest contribution to the frontonasal prominence and maxillary processes (Ribes et al., 2006; Halilagic et al., 2007). These processes driven by Aldh1a2 activity during early gastrulation are required for proper neural crest cell differentiation (Niederreither et al., 1999; Mic et al., 2010). RA regulates multiple developmental signaling pathways including; *Hox* genes, fibroblast growth factor (*Fgf8*), sonic hedgehog (*Shh*) and paired box (*Pax*) genes (Kam et al., 2012; Rhinn and Dolle, 2012; Cunningham et al., 2015). Prenatal alcohol exposure (PAE) mouse models exhibit cranium/skeletal defects and craniofacial malformations similar to RA deficiency mouse model cranial neural crest cell lineages, specifically (Sulik and Johnston, 1983; Niederreither et al., 2000; Dunty et al., 2002; Niederreither et al., 2003; Ribes et al., 2006; Anthony et al., 2010; Lipinski et al., 2012).

Despite all the strong evidence supporting PAE-induced RA-deficiency as a major etiology of the craniofacial malformations and neurodevelopmental deficits seen with FAS, this has never been directly tested in a mammalian model. To precisely induce a transient RA-deficiency in the mammalian node during early gastrulation, we generated a knock-in mouse expressing Cyp26A1 from the *Goosecoid* (*Gsc*) gene. We rationalized that if our hypothesis is true, biochemically mimicking the PAE-induced reduction of RA in the organizer (node) domain at this early developmental stage through genetic manipulation should result in many of the well-described PAE phenotypes observed in mice. The Cyp26A1 enzyme is considered the main enzyme involved in degradation of all RA isoforms (all-trans, 9-cis, 13 cis, and 18-cis). We targeted the *Cyp26A1* cassette into the *Gsc* gene locus at the same exon 2 insertion point as the well characterized *Gsc* knockout mouse (Rivera-pérez et al., 1995) by targeted homologous recombination. Expression of the exogenous Cyp26A1 will now be under the regulation of the endogenous Gsc promoter and regulatory elements and be developmentally expressed in the Node and primitive streak—E6.4-E6.9 in mouse (Blum et al., 1992)—to mimic ethanol-induced RA deficiency at the start of gastrulation. This mouse model is based on the research of co-author Dr. Abraham Fainsod, who shows that RA deficiency at the time of early gastrulation affects downstream RA signaling and phenocopies ethanol (alcohol) exposure in *Xenopus* (Yelin et al., 2005; 2007; Shabtai et al., 2018; Gur and Bendelac-Kapon, 2022). By placing the Cyp26A1 cDNA under the expression of the *Gsc* promoter we induce RA degradation in node and primitive streak and expect to see impaired downstream RA signaling and subsequent craniofacial malformations. Here we report *Gsc* ^*+/Cyp26A1*^ mice exhibit developmental defects, recapitulating many of the craniofacial malformations characteristic of PAE in mice and FASD in humans. We demonstrate that *Gsc* ^*+/Cyp26A1*^ mice—a regulated transient RA deficiency model—is a useful experimental model of early developmental origins of FASD and identifies novel therapeutic strategies for prevention.

## 2 Materials and methods

### 2.1 Generation of Gsc^+/Cyp26A1^ and Gsc^+/Cyp26A1(KO)^ mouse

**FIGURE 1:**
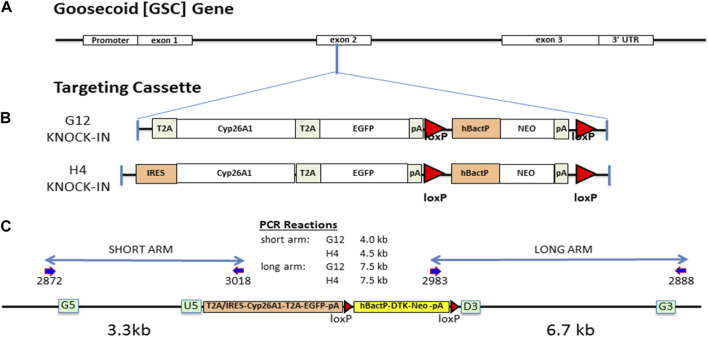
Gsc:Cyp26A1-eGFP gene targeting design and mouse derivation. **(A)** The *Gsc:Cyp26A1-eGFP* cassette was targeted to exon 2 of the endogenous *Gsc* gene by homologous recombination. **(B)** Cyp26A1 was used to catabolize RA and all RA isoforms in cells expressing Gsc. eGFP is a co-expressed fluorescent marker used to identify these cells in *in vitro* and *in vivo* studies. The cassette was constructed with two cyclin T2A peptide-bond-skipping translation elements to translate both the Cyp26A1 and eGFP gene products as individual proteins when the *Gsc* promoter was activated. Neomycin is a mammalian selection marker for gene targeting, which was later removed *in vivo* by crossing with a Cre mouse. The G12 strain uses a T2A translational element for Cyp26A1. The H4 strain uses an IRES translational element for Cyp26A1. **(C)** All targeting steps in ES cells and C57BL/6N mice were sequence validated to ensure correct recombination events and intact functional elements. Gateway recombineering sites (green box) and primers (dark blue arrows), and loxP sites (red triangle) are indicated.

We targeted a *Cyp26A1* cassette into the *Gsc* gene locus at the same exon 2 insertion point as the well characterized *Gsc* knockout mouse (Rivera-pérez et al., 1995) by targeted homologous recombination, as previously described (Consortium, 2007). Briefly, the *Gsc* genomic clone was isolated from a mouse C57BL/6N genomic library and used to create two targeting constructs containing 18 kb of genomic DNA with either a T2A-Cyp26A1-T2A-eGFP-LOX-hBacP-∆TK/NEO-LOX (G12) or an IRES-Cyp26A1-T2A-eGFP-LOX-hBacP-∆TK/NEO-LOX (H4) cassette, inserted by recombineering in a sense orientation using G5-U5 (3.3 kb) and D3-G3 (6.7 kb) *Gsc* gene homology arms (Figure 1). After electroporation in ES cells and positive/negative selection, eight targeted cell clones for each targeting vector was fully sequence validated for correct recombination, error free junctions and full insertion cassette. PCR genotyping of mice and embryos was performed from ear punch, embryonic yolk sac, or embryonic tail DNA using a forward primer located in exon 1 and two reverse primers, one located in Cyp26A1 and one located downstream in exon 2. This multiplex strategy results in two possible PCR products, one for the mutant allele (∼460bp) and one for the WT allele (∼750 bp): *Gsc* forward primer, 5′-GGG​CTA​CAA​CAG​CTA​CTT​CTA​C-3’; *Cyp26a1* reverse primer, 5′-CGT​ATT​TCC​TGC​GCT​TCA​TC-3’; *Gsc* reverse primer, 5′-TCT​CCT​GGA​AGA​GGT​TCT​CC-3’. Crispr-Cas9 was used to knock-out a 19bp portion of exon 1–2 junction of the Cyp26A1 cDNA, functionally removing Cyp26A1 mRNA expression under *Gsc* promoter activation, to produce the *Gsc* ^*+/Cyp26A1(KO)*^ strain which is functionally a *Gsc* ^*+/−*^ heterozygous mouse strain.

### 2.2 Embryoid body assay

*Gsc* ^*+/Cyp26A1*^ embryonic stem (ES) cell lines G12 and H4 were used for embryoid body (EB) experiments. When cells achieved a confluency of 80% in a 10 cm culture dish, the cells were trypsinized, washed, and underwent a 1 h incubation period without a MEF adherent layer to obtain only ES cells and remove any remaining MEFs from the cell suspension. ES cell suspensions were then collected, and 50,000 cells were placed in each well of an ultra-low density attachment 6 well plate and supplemented with 100 ng/ml of Activin A and 100 ng/ml of Basic Fibroblast Growth Factor (FGF-2), as per EB protocol (Soto-Gutierrez et al., 2007).

### 2.3 Embryo dissection and fixation

Under isofluorane anesthetic, embryos were harvested from pregnant female dams (either WT female mated with a *Gsc* ^*+/Cyp26A1*^ male or a *Gsc* ^*+/Cyp26A*^ female mated with WT male) at day E8.5, 9.5, 10.5, 11.5 or 18.5 by removal from embryonic sack. Embryonic yolk DNA was used for genotyping of early embryos. For E18.5 embryos, ∼0.5 cm of the embryo’s tail was used for genotyping. E8.5-11.5 embryos were fixed overnight (12–16 h) in 2.5 ml of freshly prepared 4% paraformaldehyde at 4°C (Anthony et al., 2010). E18.5 embryos were each placed in a 15 ml conical tube in 5 ml of 2% paraformaldehyde-2% glutaraldehyde mixture solution for fixation (required for SEM imaging and established in the Hicks lab). Embryos were kept in the fixation mixture solution until SEM imaging, within 1–2 weeks.

### 2.4 RARE-LacZ embryo expression assay

E8.5, 9.5, 10.5 or 11.5 embryos were harvested from a pregnant female *Gsc* ^*+/Cyp26A1*^ dam mated with a male *RARE-LacZ* mouse (as described above). After dissection, E8.5-11.5 embryos were processed using a β-galactosidase staining protocol (obtained from the Rivera lab; University of New Mexico) (Rossant et al., 1991). Embryos were then stained overnight at 37°C with X-gal, washed 3 consecutive times in sterile PBS and photographed. Embryos were assessed qualitatively for LacZ expression in the frontonasal prominence and for the associated shape of the frontonasal lobe. *Gsc* ^*+/Cyp26A1*^ embryos were assessed “normal” if LacZ expression and shape of the headfold region on the frontonasal lobe was indistinguishable from that of WT embryos; “mild pattern/expression change” if small reductions of LacZ expression, small changes in the bi-lobal expression pattern in the frontonasal prominence or a small squaring in the shape of the frontonasal lobe was observed; or “severe pattern/expression change” if major changes or complete loss of LacZ expression or the frontonasal lobe was observed.

### 2.5 Whole-mount *in situ* hybridization

Embryos at E8.5 were dissected from the dams, as described, and processed using a whole mount *in situ* hybridization protocol optimized for mouse embryos (Piette et al., 2008). Digoxigenin labelled RNA probes (sense and anti-sense) were prepared for *HoxA1, HoxB1* and *Snai1* using a mouse embryo cDNA library and primers containing a T7-promoter sequence to generate PCR products for downstream RNA synthesis using T7-RNA polymerase and a Digoxigenin RNA labelling kit (Piette et al., 2008). RNA probes were column purified and stored at −80°C until used. These were the primers sequences used:

*HoxA1*-T7-F1 5′-TAC​GTG​TAA​TAC​GAC​TCA​CTA​TAG​GAC​CAA​GAA​GCC​TGT​CGT​TC-3'.

*HoxA1*-R1 5′-CAG​ACA​TCT​TAA​GAC​CCG​TAA​AC-3'.

*HoxA1*-F2 5′-ACC​AAG​AAG​CCT​GTC​GTT​C-3'.

*HoxA1*-T7-R2 5′-TAC​GTG​TAA​TAC​GAC​TCA​CTA​TAG​GCA​GAC​ATC​TTA​AGA​CCC​GTA​AAC-3'.

*HoxB1*-T7-F1 5′-TAC​GTG​TAA​TAC​GAC​TCA​CTA​TAG​GTG​TGA​CAT​ACT​GCC​GAA​AGG-3'.

*HoxB1*-R1 5′-GTT​GGA​AGC​CCA​GTT​ACT​TAG-3'.

*HoxB1*-F2 5′-TGT​GAC​ATA​CTG​CCG​AAA​GG-3'.

*HoxB1*-T7-R2 5′-TAC​GTG​TAA​TAC​GAC​TCA​CTA​TAG​GTT​GGA​AGC​CCA​GTT​ACT​TAG-3'.

*Snai1*-T7-F1 5′-TAC​GTG​TAA​TAC​GAC​TCA​CTA​TAG​GAG​TTG​ACT​ACC​GAC​CTT​G-3'.

*Snai1*-R1 5′-GCC​AGA​CTC​TTG​GTG​CTT​G-3'.

*Snai1*-F2 5′-GGA​GTT​GAC​TAC​CGA​CCT​TG-3'.

*Snai1*-T7-R2 5′-TAC​GTG​TAA​TAC​GAC​TCA​CTA​TAG​GCC​AGA​CTC​TTG​GTG​CTT​G-3'.

### 2.6 Whole-mount immunohistochemistry

Embryos at E10.5 were dissected from the dams, as described, and processed using a whole mount immunohistochemistry (IHC) staining protocol (obtained from the H. Marzban lab; University of Manitoba) (Joyner and Wall 2008). IHC was performed using the mouse anti-chicken IgG monoclonal neurofilament antibody (Ab-3A10; Developmental Studies Hybridoma Bank, 1:200) followed by goat anti-mouse IgG with HRP conjugate (AP308P; EMD Millipore, 1:200).

### 2.7 Scanning Electron Microscopy and µCT

Scanning Electron microscopy (SEM) was performed on a FEI Quanta 650 FEG—a variable pressure field emission scanning electron microscope for high-resolution imaging (SE, BSE) with low-vacuum capabilities. E18.5 embryos were dissected from the dams, as described, fixed in 4% glutaraldehyde and placed horizontally into the environmental scanning electron microscope using a cradle constructed from a 15 ml falcon tube and attached to a stainless-steel stub with double sided carbon tape. E18.5 embryo (SEM) craniofacial measurements were adapted from previous publications (Anthony et al., 2010; Lipinski et al., 2012). SEM parameters: Detection Unit: LFD, Magnification: ×25, HV: 15.00kV, Chamber Pressure: 60Pa, Spot: 5.0, SEM images collected for each embryo were loaded into AMIRA (Thermo Fisher Scientific program), a quantitative measurement program, to select the area of the snout. Linear measurements and snout tracing was performed blinded to genotype, following specific anatomical craniofacial points (including the whisker pad, bigonial line, and mental region (chin). Micro Computed Tomography (µCT) was performed on the SkyScan 1,176 x-ray microtomography system equipped with a large format 11-megapixel x-ray camera. P60-75 mice were used for µCT to gather craniofacial measurements and assess malocclusions. P60-75 mouse craniofacial measurements were adapted from previous publication (Kawakami and Yamamura, 2008). µCT Parameters: Resolution: 9 um and HV: 50 kV.

## 3 Results

### 3.1 Generation of Gsc^+/Cyp26A1^ knock-in and Gsc^+/Cyp26A(KO)^ mice

To reduce the level of RA signaling at the onset of gastrulation, we inserted a Cyp26A1-eGFP expression cassette into exon 2 of the endogenous *Gsc* gene coding sequence after aa355 (Figure 1A) to drive its expression in the node of mouse embryos at early gastrulation (Rivera-pérez et al., 1995). Although, *Gsc*-null (homozygous) mice exhibit neonatal lethality due to breathing and suckling defects, *Gsc*-heterozygotes are phenotypically normal and indistinguishable from their wildtype (WT) littermates (Rivera-pérez et al., 1995; Yamada et al., 1995). Two strains were constructed differing by the translation element used for Cyp26A1 cDNA expression. The Gsc^+/Cyp26A1(G12)^ strain contains a T2A-Cyp26A1-T2A-eGFP cassette and the Gsc^+/Cyp26A1(H4)^ strain contains an IRES-Cyp26A1-T2A-EGFP cassette (Figure 1B). The IRES-Cyp26A1-T2A-eGFP construct is expected to be less efficiently translated (Kim et al., 2011), thereby producing less Cyp26A1 protein and was intended as a phenotype-severity mitigation strategy. Targeted ES clones were sequence validated across all junctions and functional elements and correct targeting of *Gsc* was confirmed by Southern blot analysis (Figure 1C and data not shown).

*Gsc* ^*+/Cyp26A1*^ mice are born fully viable, but at a reduced frequency compared to WT littermates, 0.66:1 and 0.88:1 for the *Gsc* ^*+/Cyp26A1(G12)*^ and *Gsc* ^*+/Cyp26A1(H4)*^ strains, respectively, from the expected 1:1 frequency (Figure 1D). We observed increased reabsorption rates at E8.5 in *Gsc* ^*+/Cyp26A1*^ pregnancies suggesting that some *Gsc* ^*+/Cyp26A1*^ embryos die during early-mid gastrulation; unfortunately, the conceptuses were too degraded to assess their genotype at dissection. Furthermore, the lower frequency of *Gsc* ^*+/Cyp26A1 (G12)*^ mice at weaning supports the increased resorption rate among *Gsc* ^*+/Cyp26A1*^ embryos and the strategy that the T2A-Cyp26A1-T2A-eGFP cassette might produce a more severe phenotype, such as increased embryonic lethality, compared to the H4 strain (Figure 1D). With the range of embryonic lethality for either construct at an acceptable range, we chose to move forward with the *Gsc* ^*+/Cyp26A1 (G12)*^ allele as it offered a fuller range of phenotypes and in future experiments that might test for enhancement or rescue of phenotypes. Henceforward in the paper *Gsc* ^*+/Cyp26A1 (G12)*^ will be named *Gsc* ^*+/Cyp26A1*^.

In order to confirm that Gsc^+/−^ heterozygocity was not causing the observed Gsc^+/Cyp26A1^ phenotype, the Cyp26A1-T2A-eGFP cassette had a premature stop signal in *Cyp26A1* introduced by CRISPR/Cas9 editing to effectively create Gsc^+/−^ mice. *Gsc* ^*+/Cyp26A1(KO)*^ mice were generated using the CRISPR/Cas9 system, as described previously (Ran et al., 2013). In brief, Cas9 mRNA and sgRNAs were microinjected into fertilized embryos of *Gsc* ^*+/Cyp26A1*^ mice. Deletions in the Cyp26A1-T2A-eGFP cassette allele were confirmed by Sanger sequencing analyses. *Gsc* ^*+/Cyp26A1(KO)*^ mice were born from a *Gsc* ^*+/Cyp26A1(KO)*^ x WT cross and used as a control group for phenotypic analyses with *Gsc* ^*+/Cyp26A1*^ mice. *Gsc* ^*+/Cyp26A1(KO)*^ mice are born fully viable, but at the expected Mendelian frequency compared to WT littermates (n = 82) (Table 1). This was expected as *Gsc* ^*+/Cyp26A1(KO)*^ mice are essentially *Gsc* ^*+/−*^ heterozygous KO mice which have been shown in literature to have a normal 1:1 Mendelian ratio compared to *Gsc* ^*+/+*^ mice (Yamada et al., 1995). Moreover, *Gsc* ^*+/Cyp26A1(KO)*^ mice do not show craniofacial malformations as seen in the *Gsc* ^*+/Cyp26A1*^ model, validating that the observed phenotypes are due to the *Cyp26A1-*induced RA-deficiency.

**TABLE 1:** Gsc+/Cyp26A1 are born at a reduced frequency compared to WT littermates.

Strain	Genotype	# Of offspring	HET:WT ratio
H4 (IRES)	*Gsc* ^*+/Cyp26A1*^	109	109 : 124
	WT	124	0.88:1
	Total	233	
G12 (T2A)	*Gsc* ^*+/Cyp26A1*^	140	140:211
	WT	211	0.66:1
	Total	351	
Gsc:Cyp26A1 Crispr KO	*Gsc* ^*+/Cyp26A1(KO)*^	43	43:39
	WT	39	1.10:1
	Total	82	

### 3.2 Activin A induces eGFP expression in Gsc^+/Cyp26A1^ embryoid bodies

**FIGURE 2:**
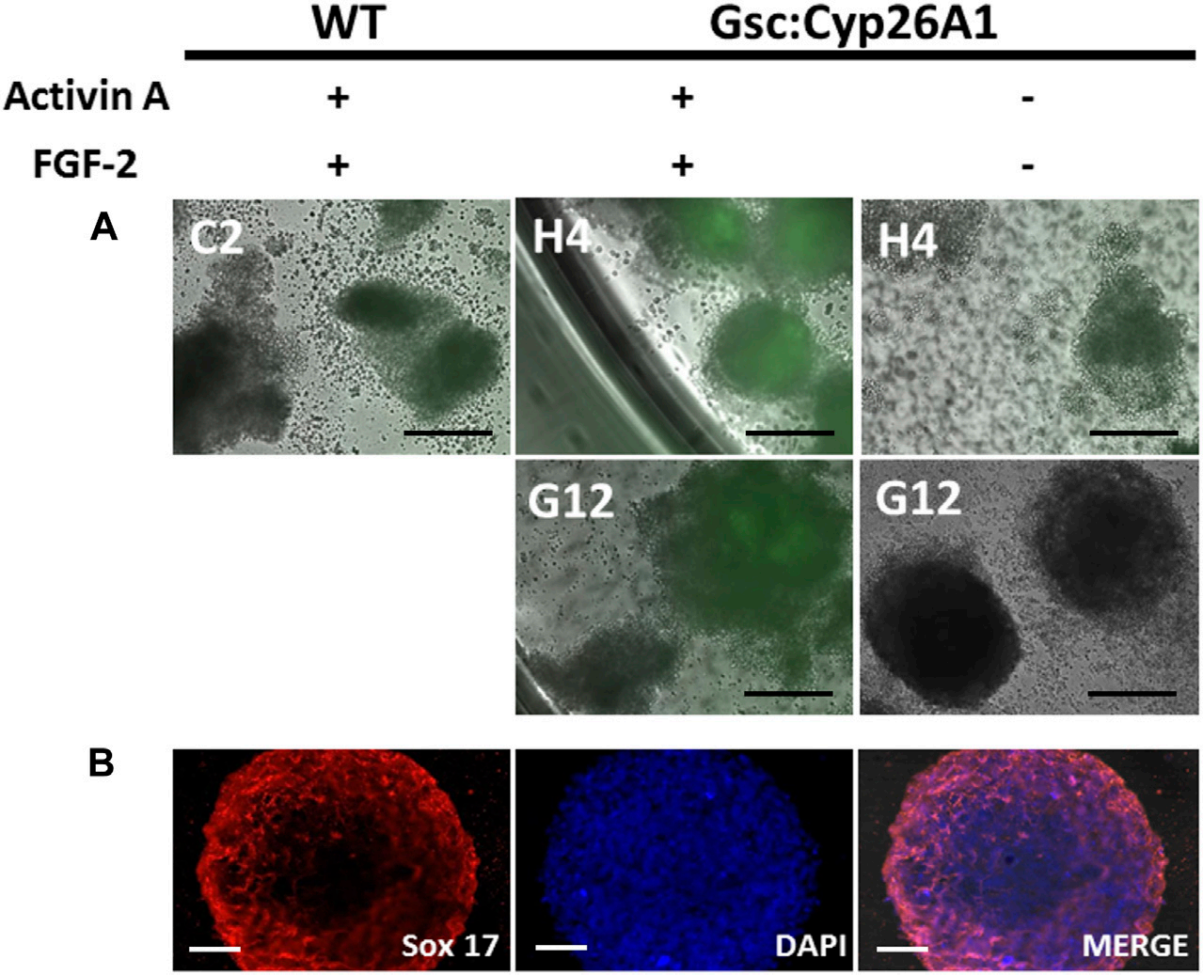
Gsc:Cyp26A1-eGFP is expressed in embryoid bodies induced with activin A and fibroblast growth factor (FGF). **(A)** Embryoid body (EB) formation was used to generate definitive endoderm germ cells *in vitro*. Activin A and FGF-2 were used to induce *Gsc* gene expression, including Cyp26A1-eGFP expression from the targeted *Gsc* allele. Results clearly demonstrate *Gsc:Cyp26A1-eGFP* is inducible under these conditions by eGFP expression in Activin A treated cells (middle panels), but not in untreated or EBs generated from wild-type ES cells (left and right panels, respectively). ES cell clones used to generate EBs are identified (C2, H4, G12). **(B)** Sox17 immunocytochemistry was performed on embryoid bodies to confirm definitive endoderm cell induction (Sox 17). Nuclear staining (DAPI) and overlay (MERGE) are shown. Scale bars: 200 um **(A)**; 40 um **(B)**.

To validate that Cyp26A1-eGFP expression is under control of the *Gsc* promoter we induced the differentiation of *Gsc* ^*+/Cyp26A1*^ ES cells by adding Activin A and FGF-2 in an *in-vitro* embryoid body (EB) assay, and looked for GFP expression as a marker of the *Gsc* gene transcription. EBs treated with Activin A initiate definitive endoderm induction, as it is in definitive endoderm where *Gsc is* expressed at the start of gastrulation (Blum et al., 1992; Soto-Gutiérrez et al., 2007). *Gsc* ^*+/Cyp26A1*^ EBs treated with Activin A and FGF-2 clearly express eGFP, compared to control EBs (Figure 2A). To verify the induction of the definitive endoderm was taking place in these Activin A treated *Gsc* ^*+/Cyp26A1*^ EBs, we confirmed the expression of *Sox17* in the EBs as a marker for definitive endoderm (Figure 2B; Iwamuro et al., 2010). These results demonstrate that Cyp26A1-eGFP is expressed in EBs under conditions known to induce the *Gsc* promoter.

### 3.3 Gsc^+/Cyp26A1^embryos exhibit headfold and other developmental malformations

**FIGURE 3:**
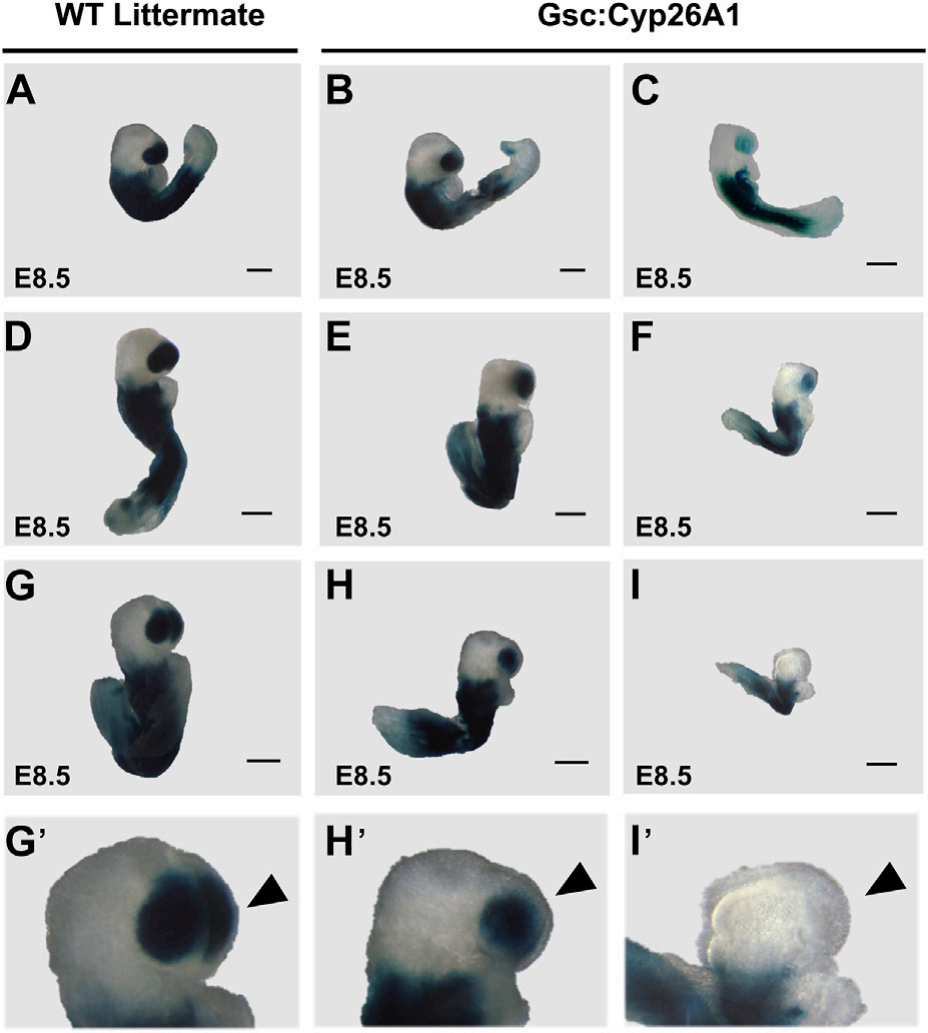
*Gsc* ^*+/Cyp26A1*^ E8.5 embryos have a reduction in RA activity/RARE-LacZ expression. **(A–I)** *Gsc* ^*+/Cyp26A1*^ mice were crossed with *RARE-LacZ* ^*+/+*^mice containing a transgene reporter for intracellular RA levels. Each row shows representative embryos from the same litter with WT, and *Gsc* ^*+/Cyp26A1*^ embryos with mild expression change or severe pattern and expression changes. *Gsc* ^*+/Cyp26A1*^ */RARE-Lac-Z* ^*+/−*^ embryos demonstrate a reduction in retinoic acid (RA) activity/RARE-LacZ expression (**B, C, E, F, H, I** lighter blue X-gal staining) in the frontonasal prominence region. WT embryos (*Gsc* ^*+/+*^ */RARE-Lac-Z* ^*+/−*^) develop proper frontonasal prominence formation and show normal RA levels **(A, D, G)**. These data show *Gsc* ^*+/Cyp26A1*^ embryos have reduced retinoic acid activity, specifically a change in RARE-LacZ expression in the frontonasal prominence. *Gsc* ^*+/Cyp26A1*^ */RARE-Lac-Z* ^*+/−*^ embryos also show aberrations in RA dependent embryonic patterning, including changes in morphology of the frontonasal prominence region (H′, I′), compared to WT siblings (G′, neural crest cell derived lineage; black arrowhead). Severe malformations and changes in RARE-LacZ expression in the frontonasal prominence region occur in approximately 16% of *Gsc* ^*+/Cyp26A1*^ */RARE-Lac-Z* ^*+/−*^ (C, F, I; Table 2). n = 10 litters, n = 30 *Gsc* ^*+/Cyp26A1*^ */RARE-Lac-Z* ^*+/−*^ embryos and n = 48 *WT/RARE-Lac-Z* ^*+/−*^ embryos. Scale bars: 200 um **(A–I)**.

To functionally validate our *Gsc:Cyp26A1-eGFP* cassette *in vivo*, *Gsc* ^*+/Cyp26A1*^ mice were crossed with the RA reporter mice (*RARE-hsp68-lacZ*) (Rossant et al., 1991). At E8.5, *Gsc* ^*+/Cyp26A1*^ */RARE-LacZ* embryos exhibit a marked reduction in RARE-LacZ expression in the frontonasal prominence, compared to wildtype embryos (Figures 3A–I). E8.5 *Gsc* ^*+/Cyp26A1*^ embryos exhibit malformations in the developing frontonasal prominence and show bilateral lobe separation compared to WT embryos (Figures 3A–I). Variation in the RARE-LacZ reporter expression in the frontonasal prominence was observed in *Gsc* ^*+/Cyp26A1*^ embryos and associated shape of the frontonasal lobe (Figure 3G’-I′-B, black arrowheads). Approximately 30% of *Gsc* ^*+/Cyp26A1*^ embryos showed a mild expression or change in RARE-LacZ expression in the headfold region and squaring in the shape of the frontonasal prominence lobes was observed, while, 16% of *Gsc* ^*+/Cyp26A1*^ embryos showed a severe expression or change—and in some cases a near complete loss - of RARE-LacZ expression in the headfold region and loss of the frontonasal prominence (Figures 3C, F, I; Table 2). The embryos with severely perturbed RARE-LacZ expression may represent the embryos that are eventually reabsorbed and explain the reduced Mendelian ratios of ∼35% and 12% loss observed at weaning in the G12 and H4 *Gsc* ^*+/Cyp26A1*^ lines, respectively (Figure 1D). In addition, E8.5 *Gsc* ^*+/Cyp26A1*^ embryos have varying degrees of severity of the head fold region and appear developmentally delayed by up to 0.5 days (E8.0–8.5), as evidenced by a smaller size, failure to turn, and a reduced number of somites compared to intralitter controls (Figures 3A–I, respectively).

**TABLE 2:** Gsc+/Cyp26A1 E8.5 embryos show changes in RARE-LacZ expression, specifically in the developing frontonasal prominence.

Embryo age	Genotype	Normal	Mild pattern/Expression change	Severe pattern/Expression change	*p*-value^a^
E8.5	WT	48	0	0	*p* = 1.4 × 10^−7^
	Gsc:Cyp26A1	16	9	5	
E9.5	WT	9	0	0	ns
	Gsc:Cyp26A1	7	0	0	
E10.5	WT	8	0	0	ns
	Gsc:Cyp26A1	6	0	0	
E11.5	WT	8	0	0	ns
	Gsc:Cyp26A1	7	0	0	

^a^ Fisher’s exact Test: ns, no statistically significant difference between groups.

**FIGURE 4:**
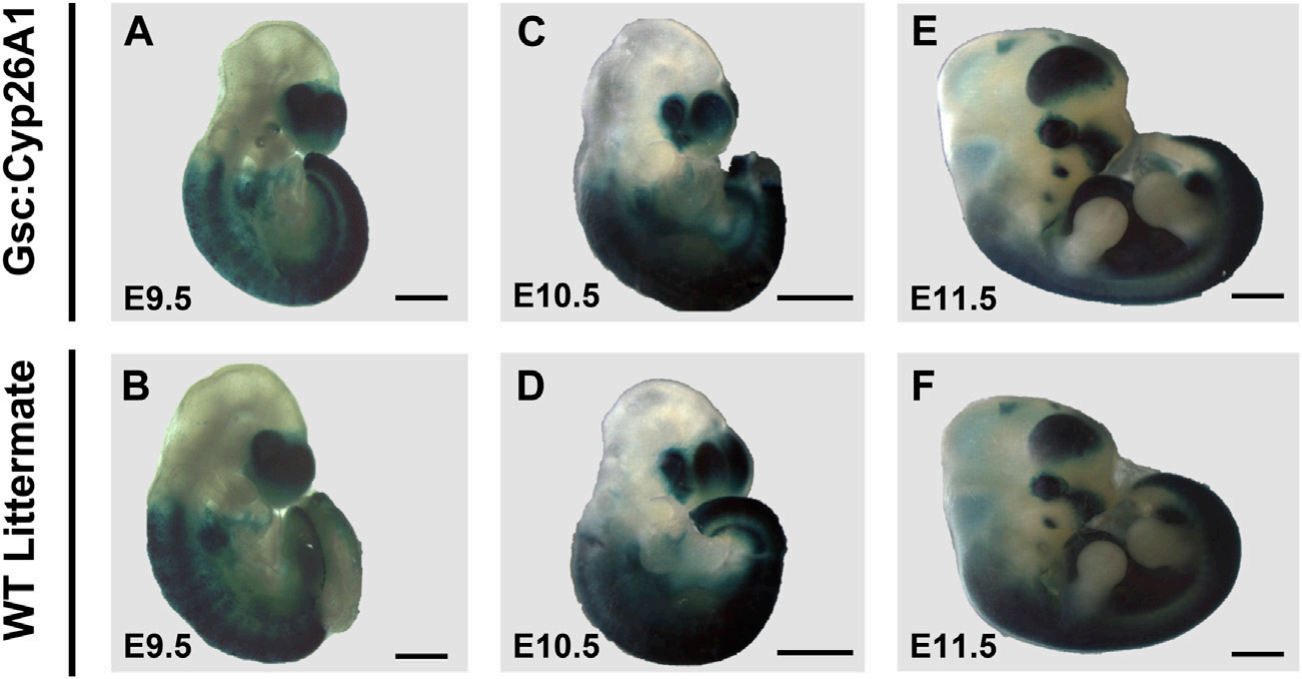
RARE-LacZ expression returns to normal in E9.5 *Gsc* ^*+/Cyp26A1*^ embryos. **(A, B)** E9.5 *Gsc* ^*+/Cyp26A1*^ embryos have a smaller head morphology compared to WT embryos, but have no distinct changes in RARE-LacZ patterning compared to WT. E10.5 and E11.5 **(E)** *Gsc* ^*+/Cyp26A1*^ embryos do not show distinct changes in embryonic patterning, gross morphology or intensity of RARE-LacZ expression in the frontonasal prominence, anterior somites, or developing trunk, as WT embryos (**D, F**, respectively). E9.5 n = 16 embryos, E10.5 n = 14 embryos, E11.5 n = 15 embryos, n = 2 (litters) per timepoint. Scale Bars: 500um **(A–D)**, 1 mm **(E, F)**.

The surviving *Gsc* ^*+/Cyp26A1*^ embryos at E9.5-11.5 do not show overt signs of developmental malformations (Figures 4A–F). E9.5 *Gsc* ^*+/Cyp26A1*^ embryos have a smaller head morphology compared to WT embryos (Figures 4A, B). E9.5 *Gsc* ^*+/Cyp26A1*^ embryos have no distinct changes in RARE-LacZ patterning, compared to WT (Figures 4A, B). E10.5 and E11.5 *Gsc* ^*+/Cyp26A1*^ embryos do not show distinct changes in embryonic patterning, gross morphology and intensity of RARE-LacZ expression in the frontonasal prominence, anterior somites, or developing trunk when compared to WT (Figures 4C–F). The RARE-lacZ pattern is normal, and comparable between *Gsc* ^*+/Cyp26A1*^ *and WT* embryos at this level of resolution, suggesting that RA signaling has recovered and is normal by E9.5 (Table 2), but it does not rule out downstream or later effects and outcomes resulting from the earlier RA deficiency.

**FIGURE 5:**
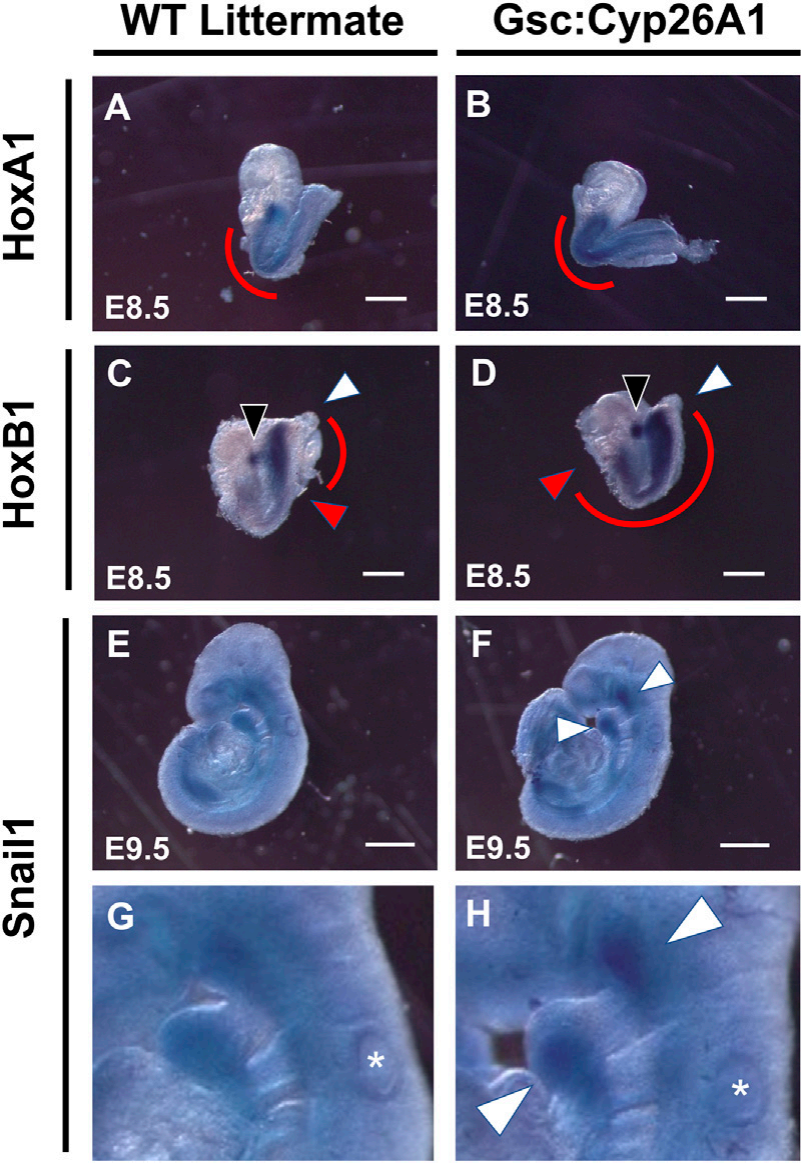
*Gsc* ^*+/Cyp26A1*^ embryos have delayed *HoxA1* and *HoxB1* transcriptional expression. *In-situ* hybridization studies show that E8.5 *Gsc* ^*+/Cyp26A1*^ embryos have increased *HoxA1* expression in the somatic (caudal to the hindbrain) and the trunk regions **(B)**, (red curve), compared to WT littermates **(A)**, (red curve). **(D)** E8.5 *Gsc* ^*+/Cyp26A1*^ embryos have increased *HoxB1* expression in rhombomere 4 (open white arrowhead) and increased expression and expansion in the somatic and trunk regions of the embryo (red curve, red arrowhead indicates rostral end of expression profile, white arrowhead indicates caudal end of expression profile), compared to WT littermates **(C)**. *Snai1* (neural crest cell lineage marker) expression pattern is similar between E9.5 *Gsc* ^*+/Cyp26A1*^ and WT littermate embryos **(E, F)**; but there remains regions (maxillary and branchial arch 1 & 2 regions, white arrows) with increased *Snai1* expression in *Gsc* ^*+/Cyp26A1*^ embryos (enlarged in **G, H**; respectively). * = otic vesicle. HoxA1 n = 15 embryos, HoxB1 n = 14 embryos, Snail1 n = 9 embryos, n = 2 (litters) per timepoint. Scale Bars: 200 um **(A–D)**, 500 um **(E–F)**.

E8.5 *Gsc* ^*+/Cyp26A1*^ embryos were further investigated for aberrant RA signaling, focusing on changes in the expression of well characterized downstream RA targets, *Hox* genes (Figures 5A–D). It has been previously established that *HoxA1* and *HoxB1* are regulated by RA through 5′ and 3’ RA response elements found in the vicinity of these genes (Ogura and Evans, 1995a; Ogura and Evans, 1995b; Dupé et al., 1997). E8.5 WT embryos exhibited normal *HoxA1* expression caudally from rhombomere 2 (r2) to the somitic mesoderm in the tail bud region (Figure 5A). *Gsc* ^*+/Cyp26A1*^ embryos exhibited increased *HoxA1* expression in the more posterior expression domain (Figure 5B). E8.5 *Gsc* ^*+/Cyp26A1*^ embryos exhibited increased *HoxB1* expression in r4 and expanded uniform *HoxB1* expression in the somitic mesoderm caudal to r4 and into the tail bud region (Figure 5D). In WT littermates, we observe *HoxB1* expression restricted to r4, without expression extending caudally from r4 (Figure 5C). The second normal expression domain in the tail bud region was also detected (Figure 5C). These observations suggest that *Hox* mediated hindbrain and somite development in *Gsc* ^*+/Cyp26A1*^ embryos is aberrant and consistent with a delay induced by RA deficiency at early gastrulation due to the *Gsc* induction of Cyp26A1. In E9.5 *Gsc* ^*+/Cyp26A1*^ and WT littermate embryos the *Snai1* expression pattern is similar (E, F), but there remains regions in the maxillary process and branchial arch 1 & 2 regions with increased *Snai1* expression in *Gsc* ^*+/Cyp26A1*^ embryos (Panel G, H, white arrows).

**FIGURE 6:**
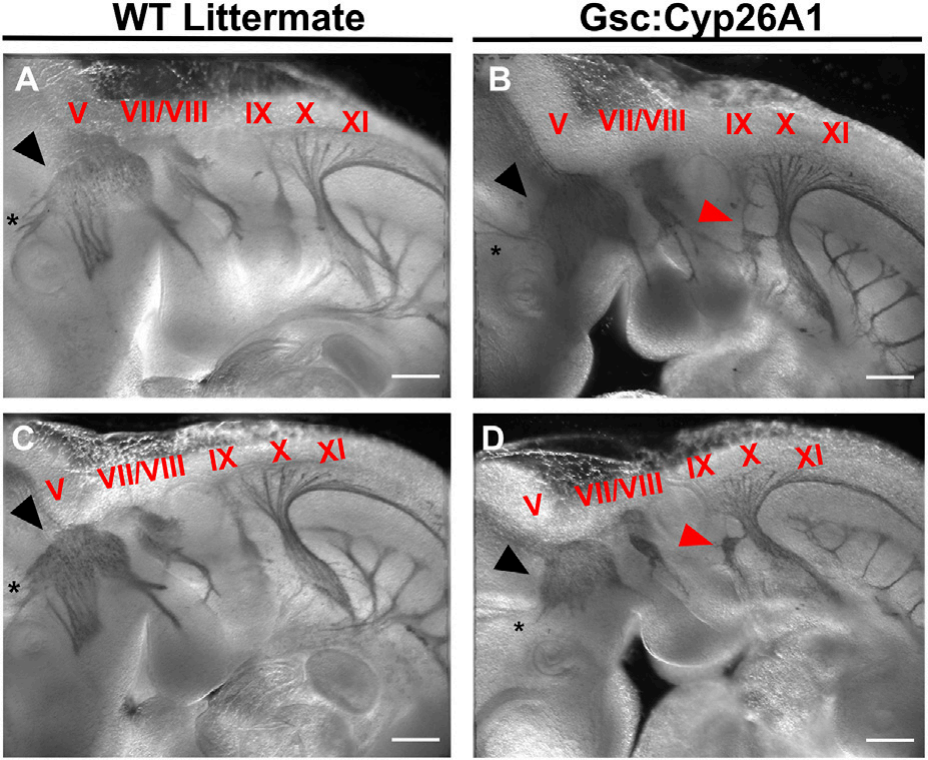
*Gsc* ^*+/Cyp26A1*^ E10.5 embryos have aberrant neural crest cell migration in the developing cranial nerves. *Gsc* ^*+/Cyp26A1*^ E10.5 embryos show a dysregulated cranial nerve patterning in the developing face and branchial arches derived from the neural crest cell lineage **(B, D)**. *Gsc* ^*+/Cyp26A1*^ E10.5 embryos have decreased neural crest cell migration in cranial nerves V (black arrows), VII, VIII, X, XI, and specifically IX (red arrows). WT Littermates demonstrate proper cranial nerve patterning in the developing face and branchial arches; and the cranial nerves are migrating as expected **(A, C)**; cranial nerves are identified by red Roman numerals). Notice that cranial nerve V does not innervate the optic vesicle in either of the *Gsc* ^*+/Cyp26A1*^ embryos, but correctly innervates the optic vesicle (black asterisk) in WT littermate embryos. These results demonstrate that the *Gsc* ^*+/Cyp26A1*^ model results in aberrant neural crest cell proliferation and migration. * = optic vesicle. Immunohistochemistry marker: Neurofilament-200 (NF-200) protein. Scale Bars: 500 um **(A–D)**.

Cranial neural crest cells (CNCC) delaminate from rhombomeres during early neurulation (E 8.5) and migrate to colonize the branchial arches. Perturbation of RA levels during gastrulation can cause malformations in the developing cranial nerves (Niederreither et al., 2003), and similar malformations are also observed in alcohol exposure during gastrulation in experimental models (Dunty et al., 2002). For these reasons, we analyzed the formation of the cranial nerves in E9.5 *Gsc* ^*+/Cyp26A1*^ embryos using the Neurofilament-200 (NF-200) protein as a marker. We observed aberrations in the Trigeminal nerve (cranial nerve V)—affecting 37.5% of *Gsc* ^*+/Cyp26A1*^ embryos—whereby the ophthalmic, maxillary, and mandibular branches do not innervate the developing optic vesicle, frontonasal prominence, and 1^st^ branchial arch, respectively (Figures 6A–D; black arrowheads and asterisks; Table 3). Additional malformations involved the development of cranial nerves VII (Facial) and VIII (Vestibulocochlear)—affecting 12.5% of *Gsc* ^*+/Cyp26A1*^ embryos—which fail to innervate the 2^nd^ branchial arch and the otic vesicle, respectively (Figures 6C, D; Table 3). *Gsc* ^*+/Cyp26A1*^ embryos also exhibit a defect in cranial nerve IX (Glossopharyngeal)—affecting 87.5% of *Gsc* ^*+/Cyp26A1*^ embryos—specifically the loss of the neural crest-derived dorsal root of this nerve, resulting in a fusion of the placode derived Glossopharyngeal cranial nerve to the Vagus (X) cranial nerve (Figures 6B, D; red arrows; Table 3). This is not the case in WT embryos which show the normal neural crest and placode derived neurofilaments of cranial nerve IX (Figures 6A, C; red arrowheads; Table 3). Beyond the connectivity defects described, E10.5 *Gsc* ^*+/Cyp26A1*^ embryos show an overall decrease in neurofilament expression level in the cranial nerves resulting in thinner dorsal root fibers innervating the frontonasal prominence and branchial arch regions in cranial nerves V-X when compared to controls (Figures 6B, D). We also observed asymmetric abnormalities in cranial nerve patterning in *Gsc* ^*+/Cyp26A1*^ E10.5 embryos when comparing the right and left sides of the same embryo (data not shown). Furthermore *Snail (Snai1),* a marker of migrating NCCs, was found to be expressed in the 1^st^, 2^nd^, and 3^rd^ branchial arches as expected of both WT and *Gsc* ^*+/Cyp26A1*^ E9.5 embryos. However, *Snai1* expression was visibly increased in *Gsc* ^*+/Cyp26A1*^ embryo branchial arches 1 and 2 compared to WT embryos (Figures 5G, H, white arrowheads). Taken together, these data indicate that the cranial NCC lineages and subsequently the developing forebrain are abnormal in *Gsc* ^*+/Cyp26A1*^ embryos, and reminiscent of RA responsive element gene knockout mouse models (Lohnes et al., 1994; Ghyselinck et al., 1997).

**TABLE 3:** Gsc+/Cyp26A1 E10.5 Embryos show Increased Incidence of Cranial Nerve Defects.

	Normal embryos	Embryos with 1 or more CN^a^ defects	Cranial nerves affected			*p*-value*
			CN V	CN VII/VII	CN IX	
WT	8	0	0	0	0	0.0014
Gsc:Cyp26A1	1	7	3	1	7	

^a^ N = cranial nerve, *Fisher’s Exact Test performed.

### 3.4 Gsc^+/Cyp26A1^ E18.5 embryos have FASD sentinel-like craniofacial phenotypes

**FIGURE 7:**
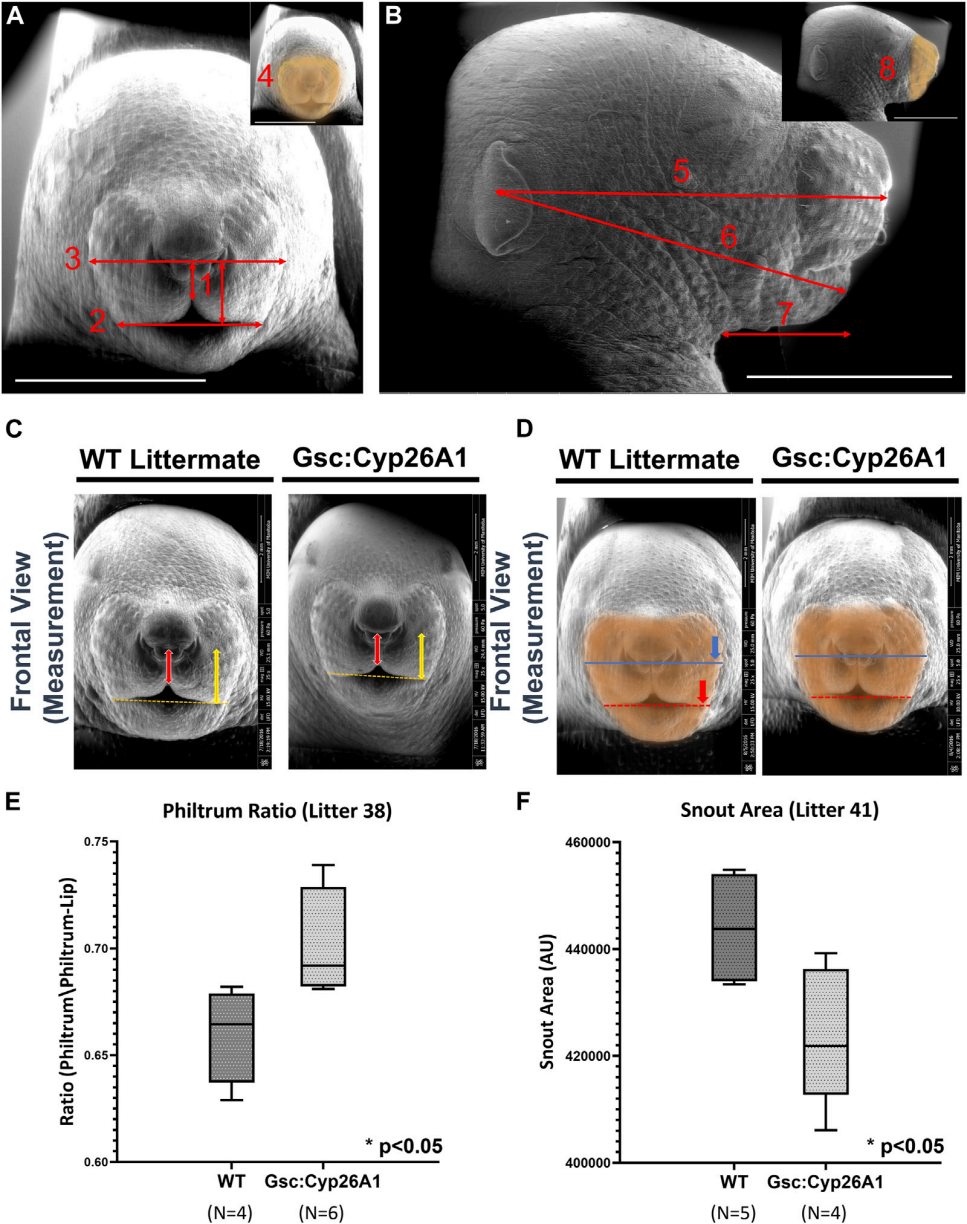
*Gsc* ^*+/Cyp26A1*^ E18.5 embryos have Fetal Alcohol Syndrome (FAS)-like craniofacial malformations. **(A)** Landmark craniofacial measurements used for E18.5 embryo facial analysis. (Adapted from Anthony et al., 2010; Lipinski et al., 2012). E18.5 embryo SEM frontal pictures were used for Philtrum-Lip Ratio (1), Bigonial Line (2), Whisker Pad (3), and Snout Area (4) quantitative measurements. **(B)** E18.5 embryo SEM side-view pictures were used for Midfacial (5), Lower facial (6), Neck to Edge of Mandible (7), and Side Snout Area (8) quantitative measurements. Inset indicates the snout area (orange highlight). **(C, E)** *Gsc* ^*+/Cyp26A1*^ E18.5 embryos have a less defined maxillary process resulting in a larger philtrum/philtrum-lip ratio compared to WT littermates (red arrow length/yellow arrow length). For comparison, WT littermates E18.5 embryos have a more defined, normal protruding maxillary formation resulting in a smaller philtrum/philtrum lip ratio. **(D, F)** *Gsc* ^*+/Cyp26A1*^ E18.5 embryos have a narrower bigonial line width (red dashed line, asterisk marks the comparative length of *Gsc* ^*+/Cyp26A1*^ bigonial line width on a WT sibling). **(D)** Whisker pad length measurement can be seen by the dark blue line, asterisk marks the comparative length of *Gsc* ^*+/Cyp26A1*^ whisker pad length on a WT sibling (Table 4). **(B, F)** The orange overlay defines a smaller snout area in *Gsc* ^*+/Cyp26A1*^ E18.5 embryos compared to WT sibling that has a larger snout area (Panel D, Table 4). Representative analysis of the most significant measurements are shown and box plots show data from litter 38 and 41, see Table 4 for litter measurement details. Scale bar = 4 mm. A t-test was used to determine statistical significance. * = *p* < 0.05.

**FIGURE 8:**
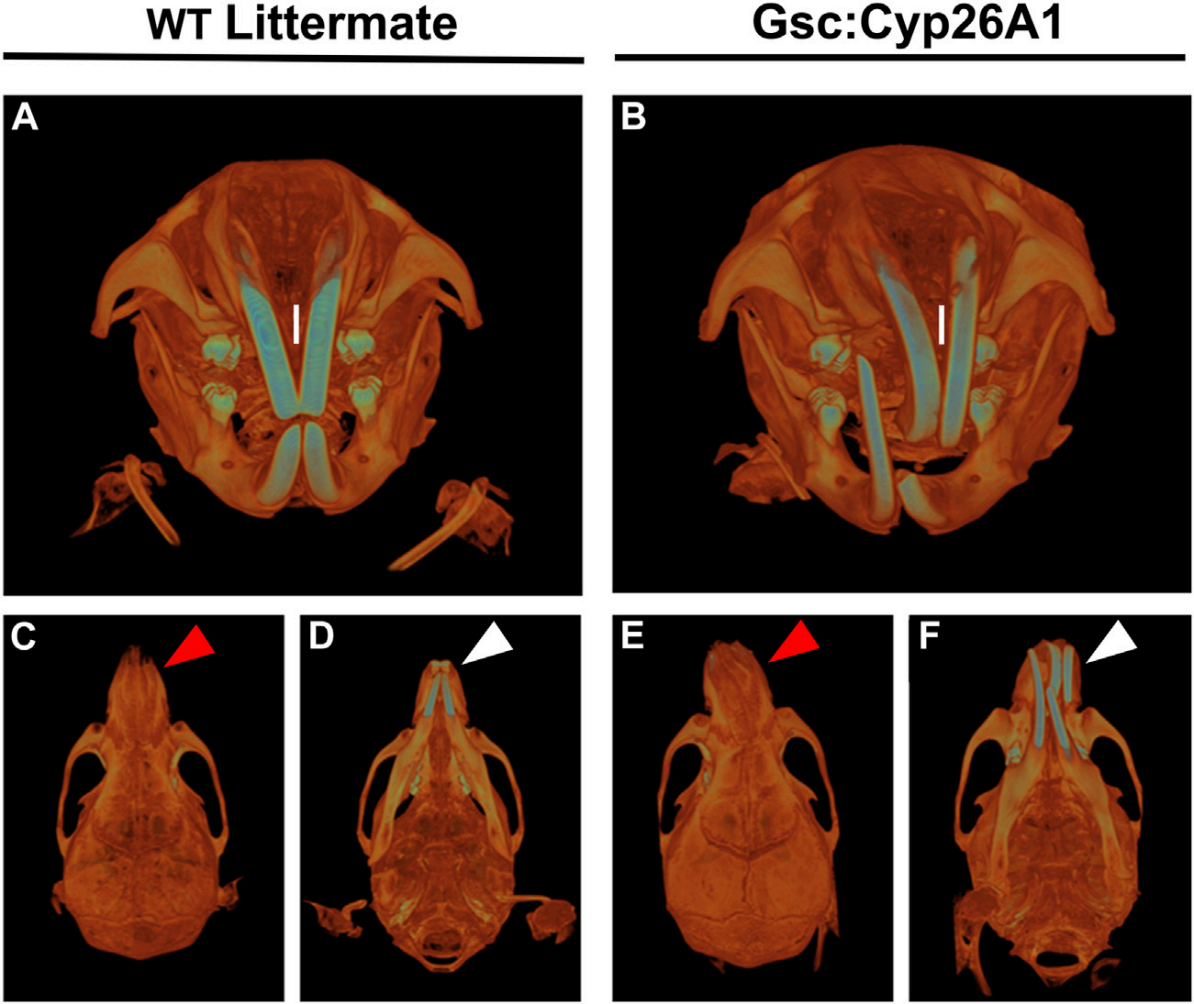
*Gsc* ^*+/Cyp26A1*^ mice have severe craniofacial malocclusions. *Gsc* ^*+/Cyp26A1*^ mice **(B)** demonstrate a curvature in the pre-maxillary process when compared to WT littermates **(A)**. *Gsc* ^*+/Cyp26A1*^ mice **(E)**, red arrow; **(F)**, white arrow do not have a curvature of the mandibular component, the mandible is straight when compared to the skull as seen in WT littermates **(C)**, red arrow; **(D)**, white arrow. The curvature to the maxillary component can be seen to impact the natural grinding of the incisors, ultimately causing a severe malocclusion in *Gsc* ^*+/Cyp26A1*^ mice (B, I = maxillary incisors); the incisors grind normally in WT littermate mice (A, I = maxillary incisors).

A specific set of craniofacial malformations, termed Sentinel Facial Features, are unique to severe forms of FASD, FAS, and they are used diagnostically (Cook et al., 2016). Individuals with FAS, those with noticeable craniofacial malformations, are found in 0.7% of the population, while individuals in the FASD spectrum make up 2%–5% of the population (May et al., 2009). We were curious if we would observe similar craniofacial malformations in our *Gsc* ^*+/Cyp26A1*^ model. We chose to use a series of landmark facial features to measure Philtrum-Lip Ratio, Bigonial Line, Whisker Pad, Frontal Snout Area, Midfacial, Lower Facial, Neck to Edge of Mandible, and Side Snout Area to determine whether our *Gsc* ^*+/Cyp26A1*^ embryos recapitulate reported PAE or other related craniofacial developmental phenotypes (Figure 7, panel A and B). We assessed 7 litters (32 *Gsc* ^*+/Cyp26A1*^ + 37 WT embryos) and found a variable penetrance in craniofacial outcomes (Table 4). To quantify the craniofacial phenotype, E18.5 *Gsc* ^*+/Cyp26A1*^ embryos were imaged using Scanning Electron Microscopy (SEM) analysis to measure the length of the bigonial line, the length of the philtrum-lip ratio, the length of the whisker pad, and the overall area of the front snout (Figures 8C–F; Table 4). The bigonial line is the horizontal line dividing the maxillary and mandibular components of the snout, while the whisker pad encompasses the maxillary portion of the snout, the nose and the philtrum (Figures 7A, D). E18.5 *Gsc* ^*+/Cyp26A1*^ embryos have a narrower bigonial line and whisker pad length (Figures 7D, F; Table 4; Panel D red and blue dashed lines, respectively). These craniofacial changes are similar to the malformations described in PAE models (Godin et al., 2010a; Anthony et al., 2010; Lipinski et al., 2012). *Gsc* ^*+/Cyp26A1*^ embryos had a significantly larger philtrum-lip ratio compared to control embryos (Figures 7C, E). Tracing of the snouts was performed following specific anatomical craniofacial points (including the whisker pad, bigonial line, and mental region (chin) to accurately trace the snout as seen in the orange overlay in Figure 7D. *Gsc* ^*+/Cyp26A1*^ embryos had a smaller frontal snout area compared to control embryos (Figures 7D, F). Both the philtrum-lip ratio and frontal snout area are new measurements, developed during this study to better assess the philtrum, maxillary and mandibular processes, as this is where the FASD sentinel facial features develop in humans. The mean for each of the 4 craniofacial measurements for both *Gsc* ^*+/Cyp26A1*^ and WT embryos in each individual litter are shown along with *p*-values for each of the intra-litter measurements (Table 4). Four craniofacial measurements: philtrum-lip ratio, bigonial line, whisker pad length and frontal snout area were found to exhibit significant (*p* < 0.05) or trending (*p* < 0.10) differences in litters analyzed. To be analyzed, a litter needed to have at least three embryos of each genotype. Larger litter sizes of 9–10 embryos were significant or trending in up to all four measurements using the t-test (e.g., litters 38 and 41, Table 4; Figure 7). Litters sizes of 7-8 embryos typically had one measurement significant or trending. It is not clear whether this drop reflects the limits of statistical analysis or the loss of more seriously impacted *Gsc* ^*+/Cyp26A1*^ embryos. Regardless, the variation in sentinel craniofacial features seen in *Gsc* ^*+/Cyp26A1*^ embryos does appear to model the spectrum observed in children with FAS, those with sentinel facial features (craniofacial malformations). Four other measurements: midfacial depth, lower-facial depth, neck to edge of mandible, and side snout area were trending in certain litters, but none of the litters were statistically significant. We assessed other craniofacial measurements that may be associated with RA deficiency or PAE but found no additional significant or trending differences in the craniofacial region between *Gsc* ^*+/Cyp26A1*^ and WT embryos. Taken together, we believe these 4 features demonstrate the effects of our transient RA deficiency model and phenocopy PAE craniofacial malformations in mice.

**TABLE 4:** Craniofacial measurements in Gsc+/Cyp26A1E18.5 Embryo Litters Compared to WT Littermate Embryos.

Litter	Bigonial line length^a^			Whisker pad length^a^			Philtrum to lip ratio			Frontal snout area^b^		
	Gsc	WT	*p*-value	Gsc	WT	*p*-value	Gsc	WT	*p*-value	Gsc	WT	*p*-value
38 (6WT:4Gsc)	3.31	3.52	0.0456	4.38	4.62	0.0518	0.701	0.663	0.0267	472	545	0.0160
41 (4WT:5Gsc)	3.06	3.23	0.0055	3.89	4.15	0.3729	0.727	0.708	0.1302	397	434	0.0216
17 (5WT:4Gsc)	3.58	3.39	0.0792	4.74	4.63	0.1904	0.610	0.711	0.0209	573	516	0.0719
16 (6WT:3Gsc)	3.70	3.84	0.2470	4.58	4.72	0.2343	0.491	0.450	0.1773	555	610	0.0806
29 (5WT:4Gsc)	3.20	3.07	0.1100	4.22	4.12	0.2581	0.666	0.679	0.3508	451	426	0.2499
35 (5WT:3Gsc)	2.89	2.90	0.2740	3.80	4.08	0.0347	0.740	0.699	0.1121	395	408	0.1605
21 (4WT:3Gsc)	3.35	3.43	0.1334	4.49	4.63	0.0927	0.638	0.622	0.3400	502	558	0.0378
			**3/7**			**3/7**			**2/7**			**5/7**

^a^ mean measure (mm) per genotype.

^b^ mean area (AU x 1,000) per genotype *p*-value: *p* < 0.05 (significant; red); *p* < 0.10 (trending; blue).

### 3.5 Gsc^+/Cyp26A1^ mice develop craniofacial maxillary malocclusions

**FIGURE 9:**
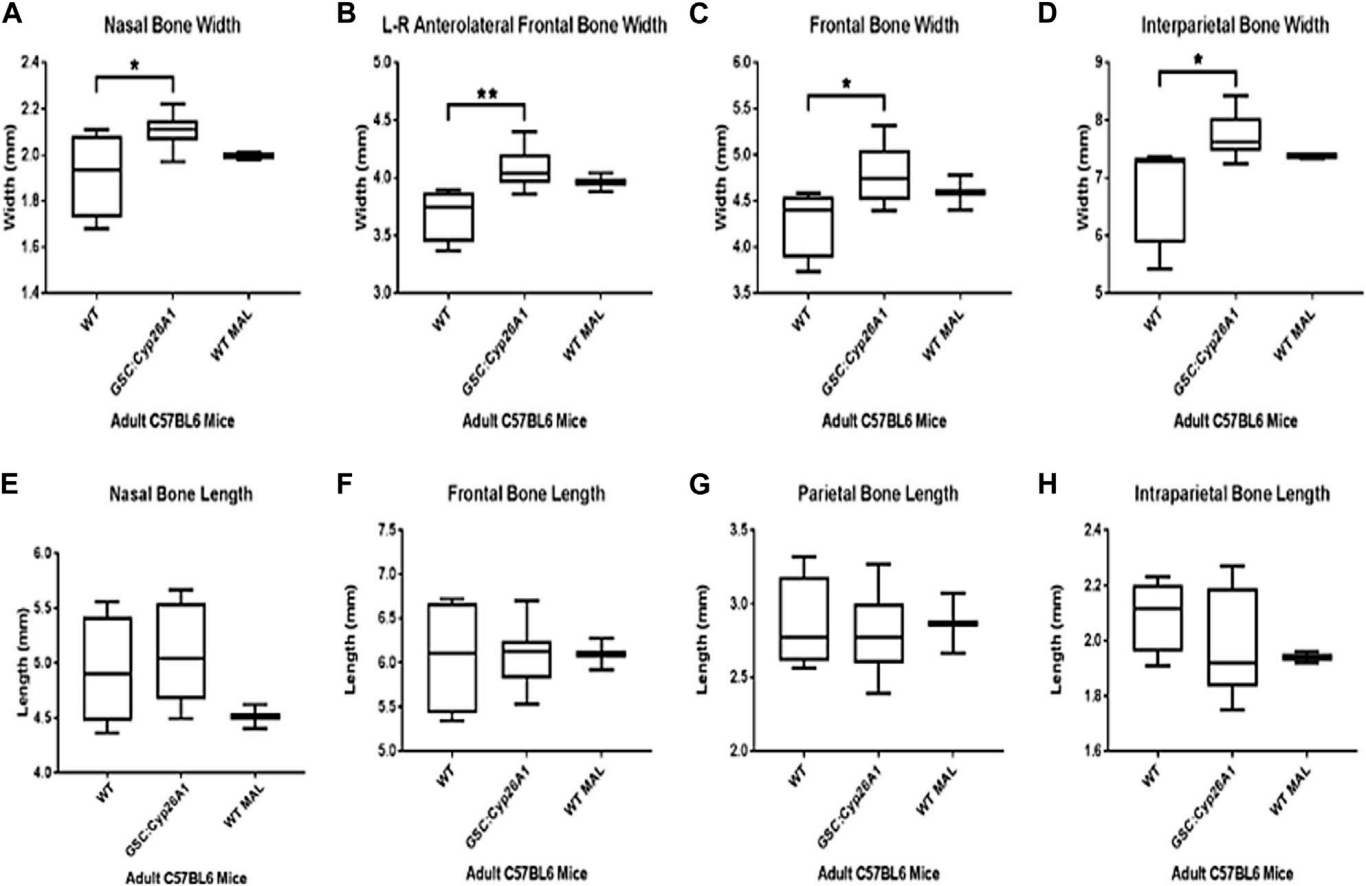
*Gsc* ^*+/Cyp26A1*^ mice have a wider cranium compared to WT littermates. P60 *Gsc* ^*+/Cyp26A1*^ mice show a statistically significant wider cranium in all 4 major skull region width measurements: Nasal Bone **(A)**, L-R Anterolateral Frontal Bone **(B)**, Frontal Bone **(C)**, and Intraparietal Bone Widths **(D)**. *Gsc* ^*+/Cyp26A1*^ mice do not demonstrate a statistically significant difference in the 4 major skull region length measurements: Nasal Bone **(E)**, Frontal Bone **(F)**, Parietal Bone **(G)** and Intraparietal Bone lengths **(H)**. *Gsc* ^*+/Cyp26A1*^ mice, n = 10; C57BL/6N mice, n = 5; WT with spontaneous malocclusions, n = 3. A t-test was used to determine statistical significance. *p < 0.05, **p < 0.01.

*Gsc* ^*+/Cyp26A1*^ mice develop overtly normal through adulthood, with one notable exception: between days 60 and 75 postnatally (PN60-75) they developed an irregular alignment of the teeth known as severe craniofacial maxillary malocclusions, caused by uneven teeth wear or a misalignment of the jaw (Figures 8B, E, F) (Diagnosis | Severe prognathic malocclusion, 2007). We observed craniofacial malocclusions in 11 of 88 *Gsc* ^*+/Cyp26A1*^ mice, a significant result when compared to WT littermates (12.5% frequency; *p* < 0.001; Figures 9B, e, F; Table 5). Malocclusions in WT C57BL/6N mice are rare with a spontaneous incidence of 0.04% in the Jax Laboratory colony and slightly higher in our own C57Bl/6N breeding colony at 0.32% (*p* < 0.001; C57BL/6N In-House colony, University of Manitoba; Table 5). To further characterize the jaw, denture, and craniofacial phenotype of the *Gsc* ^*+/Cyp26A1*^ mice and WT littermates were performed by µCT-based analysis and measurements. *Gsc* ^*+/Cyp26A1*^ mice have pre-maxillary twisting resulting in misaligning of the maxillary and mandibular incisors, specifically an anterior transverse crossbite (Tsukamoto et al., 2010) (Figure 8). Pre-maxillary malocclusions occurred at equal rates to the left or right of cranium center and the malocclusions were never mandibular in origin. In contrast, no WT littermate had craniofacial malocclusions or pre-maxillary twisting (Figures 8A, C, D; Table 5). We were able to assess wild-type C57BL/6N In-House colony mice that developed spontaneous malocclusions at 4–10 weeks (Table 5). Interestingly, all were found to have malocclusions resulting from mandibular twisting, not pre-maxillary twisting as found in *Gsc* ^*+/Cyp26A1*^ mice.

**TABLE 5:** Frequency of severe craniofacial malocclusions in Gsc+/Cyp26A1 mice.

	*Gsc* ^*+/Cyp26A1*^ ^a^	WT littermates^b^	C57Bl/6N In house colony^a^
G12	7/44 (15.9%)	0/70 (0.0%)	11/3733 (0.32%)
H4	4/44 (9.1%)	0/50 (0.0%)	
Total	11/88 (12.5%)***	0/120 (0.0%)	11/3733 (0.32%)

^a^ C57Bl/6N Background

^b^ University of Manitoba C57Bl/6N Mouse Facility Colony.

*** *p*-value <0.001 when compared to WT littermates and In House Colony (Fisher’s exact test performed).

µCT imaging allowed us to further characterize the craniofacial malformations in *Gsc* ^*+/Cyp26A1*^ mice. The *Gsc* ^*+/Cyp26A1*^ mice exhibited a significantly wider L-R anterolateral bone, overall width of the cranium, the nasal bone, the frontal bone, and the interparietal bone compared to WT littermates (*p* < 0.05) and were similar in overall width measurements to C57BL/6N In-House colony malocclusion mice (Figures 9A–D, respectively). Interestingly, *Gsc* ^*+/Cyp26A1*^ mice did not have a significant increase in the overall length of the cranium, length of the nasal bone, frontal bone, parietal bone, and interparietal bone and were similar to WT littermate mice or C57BL/6N In-House colony malocclusion mice (Figures 9E–H, respectively). We extended the initial cranium µCT width and length measurements with 11 maxillary and mandible length measurements, using the same three cohorts of mice (Supplementary Figure S1). Of those 11 length measurements, *Gsc* ^*+/Cyp26A1*^ mice have a significantly larger upper incisor height (*p* < 0.001), as expected compared to WT littermate mice, due to the pre-maxillary twisting causing malocclusion (Supplementary Figure S1). The maxillary and mandible length measurements suggests that the *Gsc* ^*+/Cyp26A1*^ mice are indeed in Class I malocclusion, causing an anterior transverse crossbite, as the maxillary and mandibular lengths were similar between all three cohorts of mice. Compared to a Class II malocclusion scenario “overbite”, where the maxilla is longer in length than the mandible, or Class III malocclusion scenario “underbite”, where the mandible is longer in length than the mandible, in either of those situations the incisors would over grow and not be gradually worn down and can impinge the soft tissues of the oral cavity and snout. In some cases of class III malocclusion, a cross-bite can occur, but the mandible would have to be longer in length than the maxilla which is not the case in *Gsc* ^*+/Cyp26A1*^ mice (Supplementary Figure S1).

## 4 Discussion

In the present study we took advantage of our biochemical and developmental understanding of FASD to establish a genetic mouse model that recapitulates many of the developmental malformations characteristic of prenatal ethanol exposure during embryogenesis. Using our *Xenopus laevis* FAS model, we demonstrated that biochemically, ethanol competes for the retinaldehyde dehydrogenase (Aldh1a2) enzymatic activity, causing a reduction in RA biosynthesis (Kot-Leibovich and Fainsod, 2009; Shabtai et al., 2018). This reduction in RA signaling affects the expression of retinoic acid downstream targets during gastrulation, and results in a high incidence of developmental malformations such as microcephaly, reduced axial length, and craniofacial defects in embryos exposed to ethanol (Yelin et al., 2005; 2007; Shabtai et al., 2018; Shukrun et al., 2019; Gur and Bendelac-Kapon, 2022). These malformations are characteristic of other experimental model embryos exposed to ethanol and of individuals affected by FASD (Petrelli et al., 2017; Shabtai and Fainsod, 2018; Petrelli et al., 2019). RA deficiency in mammalian embryos causes developmental malformations impacting all germ layer lineages including: craniofacial and brain malformations, limb deformities, organ defects, and neural crest cell migration and proliferation anomalies, among others (Sulik et al., 1988; Niederreither et al., 2003; Cunningham et al., 2011; Lee et al., 2012). In this study, we describe a genetic mouse model that induces a transient RA deficiency in the node during gastrulation which recapitulates the PAE-induced phenotypes in mice and suggests a molecular etiology for the craniofacial malformations seen in children with FASD.

### 4.1 *Gsc* ^*+/Cyp26A1*^ induces an early developmental RA deficiency

*Gsc* ^*+/Cyp26A1*^ mice were designed to create a genetic model that would induce a transient, spatially and temporally regulated RA deficiency during early gastrulation in the node, mimicking the biochemical effect of acute PAE at E6.5—E7. We observed frontonasal prominence and head fold malformations as early as E7.5. The frontonasal prominence region is important for proper craniofacial morphogenesis (Ribes et al., 2006) and shares some of the same signaling pathways needed for proper forebrain development, all of which require tightly regulated RA signaling (Dupé and Pellerin, 2009; Kam et al., 2012; Rhinn and Dolle, 2012). RA is produced in the frontonasal prominence region by Aldh1a2 and Aldh1a3, and loss of either enzyme results in craniofacial malformations (Ribes et al., 2006) or loss of RA signaling in the frontonasal prominence like we report here in the *Gsc* ^*+/Cyp26A1*^ mice and others in *Aldh1a2* ^*−/−*^ mutant mice (Niederreither et al., 2003; Ribes et al., 2006). Changes in RARE-LacZ expression have not been visualized in the frontonasal prominence of PAE embryos, likely a complication of the low penetrance of FASD-like phenotypes in murine models of alcohol exposure. Nevertheless, malformations of the frontonasal prominence have been extensively characterized (Sulik et al., 1981; Sulik and Johnston, 1983), as recapitulated in our *Gsc* ^*+/Cyp26A1*^ mouse model.

*Gsc* ^*+/Cyp26A1*^ mice have an increased incidence of early embryonic lethality, a phenotype shared in both RA deficiency and PAE mouse models. A severe loss of early RA biosynthesis in the form of *Aldh1a2* knockout results in early embryonic lethality (Niederreither et al., 1999; Niederreither et al., 2003). Similarly, embryonic lethality has been observed in *Rdh10*, double *RXR* or *RAR* α/β, α/γ, and β/γ mutants (Lohnes et al., 1994; Mark et al., 2006), stillbirths in *RXRα* (Mascrez et al., 2009), and neonatal lethality in *Aldh1a3* mutant mice (Dupé et al., 2003; Molotkova et al., 2007; Sandell et al., 2007). PAE in mice can result in embryonic lethality (Sulik et al., 1981; Sulik and Johnston, 1983), while PAE in humans is also associated with an increased risk of miscarriages, stillbirths, and sudden infant death syndrome (Bailey and Sokol, 2011; Andersen et al., 2012). Incidence of embryonic lethality (miscarriage) and stillbirths in FASD and in RA deficiency animal models further support a common signaling etiology between these disorders.

The *Gsc* ^*+/Cyp26A1*^ embryos are developmentally delayed when compared to WT embryos within the same litter as determined by the RARE-lacZ expression profile in the frontonasal prominence. This delayed RA signaling pattern phenotype has been described in *Aldh1a2* ^*−/−*^ and *Aldh1a3* ^*−/−*^ mutant mice (Ribes et al., 2006; Halilagic et al., 2007; Molotkova et al., 2007). The RA signaling developmental delay in *Gsc* ^*+/Cyp26A1*^ embryos was further supported by analyzing the *HoxA1* and *HoxB1* expression patterns. *HoxA1* is more intense and more rostral, while by E8.5, caudal to the rhombencephalon it should be gradually declining from r6 (Makki and Capecchi, 2011). In support, *Gsc* ^*+/Cyp26A1*^ embryos exhibited increased *HoxB1* expression in r4, indicative of a later onset of *HoxA1* expression due to earlier perturbation of RA. By this stage, *HoxB1* expression should have regressed to the body region, suggesting an RA deficiency mediated delay in *HoxA1 and B1* expression (Deschamps and van Nes, 2005). RA has been shown to be a required signal for proper *Hox* gene regulation and aberrant RA signaling impairs hindbrain and mesodermal patterning, (Lohnes et al., 1994; Niederreither et al., 1999; Niederreither et al., 2000; Halilagic et al., 2007; Rhinn and Dolle, 2012). Interestingly, HoxA1 is required to activate *Aldh1a2* and subsequently *HoxB1* expression (Vitobello et al., 2011). Therefore, we could hypothesize that regulated ectopic expression of Cyp26A1—leading to early gastrula RA deficiency in *Gsc* ^*+/Cyp26A1*^ mice—causes a delayed or weaker activation of *HoxA1* and *HoxB1* which in turn, hampers the expression of *Aldh1a2* further reducing the production of RA The regulated nature of our RA knockdown model makes the effect of the Cyp26A1 activity transient allowing for recovery.

Defective cranial nerve growth and connectivity is commonly seen in *Gsc* ^*+/Cyp26A1*^ embryos due to reduced RA signaling during early gastrulation. These embryos exhibited an overall reduction in ventral neurofilament-positive fibers compared to their WT littermates. The Trigeminal nerve (cranial nerve V) was affected along its three branches; ophthalmic, maxillary, and mandibular. This aberrant Trigeminal cranial nerve *Gsc* ^*+/Cyp26A1*^ phenotype is reminiscent of *HoxA1* and *HoxB1* knock-out mice as well as the *LgDel* mouse model of 22q11.2 Deletion Syndrome (Gavalas et al., 1998; Karpinski et al., 2014). Interestingly, when retinoic acid expression was altered in the *LgDel* model—through delivery of exogenous RA (increase in RA expression) or by creating a Raldh2^+/−^ x *LgDel* model (decrease in RA expression)—even more severe craniofacial developmental malformations than found in the *LgDel* model (Maynard et al., 2013). These observations suggest that a change in RA levels—whether an increase or decrease—can substantially alter downstream retinoic acid signaling and produce a wide array of phenotypic variation as seen in Vitamin A Deficiency Syndrome and 22q11.2 Deletion (DiGeorge/VeloCardioFacial) Syndrome (Vermot et al., 2003). *Gsc* ^*+/Cyp26A1*^ embryos also show aberrant patterning of cranial nerves VII and VIII based on neurofilament staining, a phenotype reminiscent of *HoxA2* knockout mice (Gavalas et al., 1997). In addition, by E10.5, *Gsc* ^*+/Cyp26A1*^ embryos had fused IX-X cranial nerves with no dorsal cranial nerve IX fibers, a developmental malformation also found in *Nav2* mutant mice (Mic et al., 2010)*.* Importantly*, Nav2* is a known RA-responsive gene (Muley et al., 2008). Null *Hoxa3* embryos exhibit both the a reduction or loss of dorsal root fibers in cranial nerves V, VII, and VIII and a cranial nerve IX/X fusion (Watari et al., 2001; Chojnowski et al., 2016). Aberrant cranial nerve V, VII, and IX phenotypes, and reduction or loss of dorsal root fibers and cranial nerve IX/X fusion have been also described in PAE mouse embryos exposed to alcohol during gastrulation (Van Maele-Fabry et al., 1995; Dunty et al., 2002; Mooney and Miller, 2008). These observations suggest transient ectopic expression of Cyp26A1 in *Gsc* ^*+/Cyp26A1*^ mice is sufficient to dysregulate cranial nerve development.

### 4.2 RA deficiency phenocopies the craniofacial malformations induced by alcohol

Individuals with FAS, the most severe form of FASD, have sentinel facial features and represent 10%–15% of children in the spectrum (May et al., 2009). Here we report *Gsc* ^*+/Cyp26A1*^ embryos develop similar craniofacial malformations with a comparable variable penetrance. E18.5 *Gsc* ^*+/Cyp26A1*^ embryos have a larger philtrum to lip ratio, a smaller frontal snout area, smaller whisker pad length, and a smaller bigonial width compared to WT littermates. More importantly, we demonstrated that RA deficiency at gastrulation in the *Gsc* ^*+/Cyp26A1*^ model produced craniofacial malformations resembling the effects of PAE at gastrulation (Sulik and Johnston, 1983; Godin et al., 2010a; Anthony et al., 2010; Lipinski et al., 2012). A smaller bigonial width has been shown to be a significant characteristic measurement in models where ethanol was administered acutely at gastrulation (E7.0-E8.5) (Anthony et al., 2010; Lipinski et al., 2012) demonstrating that RA deficiency at gastrulation phenocopies this PAE phenotype. Additionally, our study found two new statistically significant diagnostic measurements, philtrum to lip ratio and the frontal snout area, to further demonstrate changes in maxillary process formation in E18.5 *Gsc* ^*+/Cyp26A1*^ embryos. The philtrum to lip ratio measurement takes into consideration the length of the philtrum over the length of the maxillary component of the snout (bottom of the nose to the top of the mandible; lip - ratio) and is generally larger in *Gsc* ^*+/Cyp26A1*^ than WT littermates. The maxillary region in E18.5 *Gsc* ^*+/Cyp26A1*^ embryos is clearly affected; it is narrower and shorter in *Gsc* ^*+/Cyp26A1*^ embryos, as seen in PAE models (Sulik and Johnston, 1983; Parnell et al., 2009). The frontal snout area measurement further demonstrates that the entire snout of E18.5 *Gsc* ^*+/Cyp26A1*^ embryos—which includes the maxillary process region—is smaller in the genetically manipulated embryos reaching statistical significance in 5 out 7 litters. While the frontal snout area measurement is novel, this phenotype has been seen qualitatively in early gastrulation PAE mouse models (Sulik and Johnston, 1983; Godin et al., 2010). Taken together, our *Gsc* ^*+/Cyp26A1*^ model demonstrates that craniofacial malformations affecting the maxillary region are the result of RA deficiency and they phenocopy the PAE craniofacial malformations.

*Gsc* ^*+/Cyp26A1*^ mice exhibit a relatively high incidence of maxillary malocclusions which resemble those found in an early gestational PAE mouse model (Kaminen-Ahola et al., 2010). It has also been well documented that Vitamin A deficiency—on its own, RA signaling reduction, RAR loss-of-function, biosynthesis reduction, or downstream transcriptional target mutation—causes maxillary malocclusions in murine models and also in humans with VAD (Lohnes et al., 1994; Dupe et al., 2003; Niederreither et al., 2003; Kurosaka et al., 2017). Maxillary malocclusions, specifically Class I anterior transverse malocclusions, are not commonly reported and are usually overlooked as a co-morbidity caused by maternal PAE. But a few studies have shown that children with FASD do indeed have Class I, II, and III malocclusions with underdeveloped maxillary, anterior crowding, and increases of open bite (Church et al., 1997; Astley and Clarren, 2001; Naidoo et al., 2006). Recently, (Blanck-lubarsch et al., 2019), have shown that children with FASD present with anterior maxillary and transverse plane malocclusions, specifically crossbites or edge-to-edge bites which were found in *Gsc* ^*+/−*^ mice. *Gsc* ^*+/−*^ mice have not been documented to develop craniofacial maxillary malocclusions (Rivera-pérez et al., 1995). Interestingly, E0.5 to E8.5 first-trimester PAE-treated mice were shown to have wider (but not longer) cranium measurements and pre-maxillary malocclusions compared to non-PAE mice (Kaminen-Ahola et al., 2010). Our *Gsc* ^*+/Cyp26A1*^ model produced pre-maxillary malocclusions analogous to the Kaminen-Ahola *et al.* (2010) early gestation PAE model, which affected the cranial neural crest cells during gastrulation (E6.5–8.5). It is important to emphasize the *Gsc* ^*+/Cyp26A1*^ cassette is active at early gastrulation (∼E6.5-E7.0), a very short window of developmental time, but yet produced the maxillary malocclusions as seen in a chronic PAE mouse model. Therefore, early gastrulation (∼E7.0) PAE mouse models should produce maxillary malocclusions, unfortunately, many PAE studies do not study adult mice for craniofacial malformations, let alone maxillary malocclusions. Furthermore, cleft-lip and palate, maxillary hypoplasia, and tooth malformations are found—within the pre-maxillary region—in over 30% of individuals with FASD (Popova et al., 2016), a staggering figure. While such malformations are not commonly viewed as a co-morbidity of FASD, they can be used as another facial feature for assistance in FASD diagnosis.

### 4.3 PAE-induced retinoic acid deficiency can explain neurodevelopmental and craniofacial malformations seen in FASD

FASD is a spectrum of conditions arising as a result of prenatal alcohol exposure in humans. Developmental phenotypic similarities between the human and animal models of prenatal alcohol exposure are extensively observed. The *Gsc* ^*+/Cyp26A1*^ mouse model demonstrates many of the craniofacial malformations shared between RA deficiency and PAE mouse models and in human children with FASD. It is important to emphasize that our model can recapitulate neurodevelopmental and FAS-like sentinel craniofacial features following a restricted, binge like, spatial-temporal restricted activation of the *Gsc* ^*+/Cyp26A1*^ cassette during gastrulation and the resulting transient reduction in RA signaling. It is likely that taking a genetic approach to biochemically mimic the PAE-induced RA deficiency resulted in a higher penetrance of the PAE phenotype we observed, mitigating the common issues associated with PAE models due to a broad variation in dosage, duration, and timing of alcohol exposure on individual developing embryos (Petrelli et al., 2017).

Taken together, our data provide *in vivo* evidence in a mammalian model that strongly supports RA deficiency as a major molecular etiology of the craniofacial and neurodevelopmental malformations associated with FASD outcomes, as we previously supported in our *Xenopus* FAS model (Fainsod et al., 2020). Our *Gsc* ^*+/Cyp26A1*^ mouse model recapitulates nearly all PAE phenotypes, yet there is one common finding in *Xenopus* and mouse PAE models, microcephaly, which we have not seen in our genetic model. To address the missing phenotype we must remind ourselves that the *Gsc* ^*+/Cyp26A1*^ model is an acute RA deficiency at early gastrulation, not a chronic or repeated acute PAE exposure as seen in studies which show microcephaly as a PAE phenotype (Parnell et al., 2009; Godin et al., 2010a; Lipinski et al., 2012). Instead, our acute RA deficiency model resembles a chronic PAE exposure as seen in studies which show craniofacial widening as a PAE phenotype (Kaminen-Ahola et al., 2010). These findings suggest that Vitamin A supplementation may significantly reduce or prevent FASD outcomes in cases of high risk for PAE. Further studies exploring the protective effects of Vitamin A in PAE mouse models will need to be completed before a therapeutic clinical application of Vitamin A can be implemented. Our *Gsc* ^*+/Cyp26A1*^ mouse model should lead to more effective diagnostic and prevention strategies for FASD, hopefully reducing the burden of this disorder on children, their families, and society.

## Data Availability

The original contributions presented in the study are included in the article/supplementary material, further inquiries can be directed to the corresponding author.

## Supplementary material

Supplemental Figure S1
*Gsc* ^*+/Cyp26A1*^ P60 mice have large upper incisors, but do not have any length or height variations in the cranium or mandible regions. Box plots for all height and length craniofacial landmark features are shown, as indicated. *Gsc* ^*+/Cyp26A1*^ P60 mice were statistically different for only one measurement: the upper incisor height (upper right panel, P < 0.001). Trending results in the inferior incisor axis, posterior cranial height and mandible axis were observed in *Gsc* ^*+/Cyp26A1*^ mice. *Gsc* ^*+/Cyp26A1*^ mice, n = 10; C57BL/6N mice, n = 4; WT with spontaneous malocclusions, n = 3. ***P < 0.001.
