# Supplementary figures and images for: Genetically programmed retinoic acid deficiency during gastrulation phenocopies most known developmental defects due to acute prenatal alcohol exposure in FASD
Source: Front Cell Dev Biol. 2023 Jun 16;11:1208279. doi: 10.3389/fcell.2023.1208279 (PMC10311642; doi:10.3389/fcell.2023.1208279)

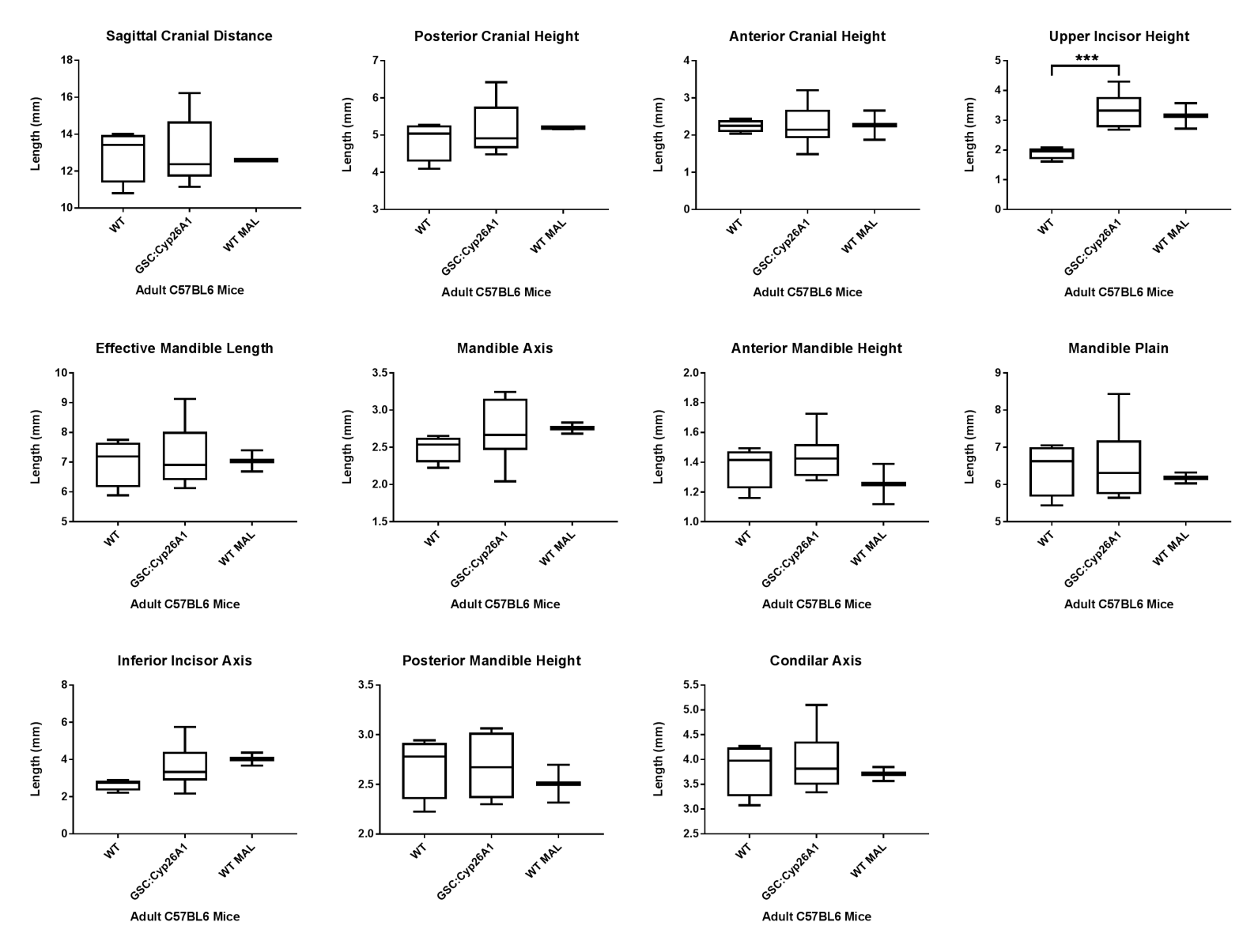

Supplement: Supplementary file 1 [file Image1.TIF]
